# Both Creatine and Its Product Phosphocreatine Reduce Oxidative Stress and Afford Neuroprotection in an *In Vitro* Parkinson’s Model

**DOI:** 10.1177/1759091414554945

**Published:** 2014-10-17

**Authors:** Mauricio Peña Cunha, Maria D. Martín-de-Saavedra, Alejandro Romero, Javier Egea, Fabiana K. Ludka, Carla I. Tasca, Marcelo Farina, Ana Lúcia S. Rodrigues, Manuela G. López

**Affiliations:** 1 Facultad de Medicina, Instituto Teófilo Hernando, Universidad Autónoma de Madrid, Spain; 2 Departamento de Farmacología y Terapéutica, Facultad de Medicina, Universidad Autónoma de Madrid, Spain; 3 Departamento de Bioquímica, Universidade Federal de Santa Catarina, Centro de Ciências Biológicas, Florianópolis, SC, Brazil; 4 Department of Physiology, Northwestern University, Feinberg School of Medicine, Chicago, IL, USA; 5 Departamento de Toxicología y Farmacología, Facultad de Veterinaria, Universidad Complutense de Madrid, Spain; 6 Instituto de Investigación Sanitaria Hospital de la Princesa, Madrid, Spain; 7 Department of Pharmacy, Universidade do Contestado, Canoinhas, SC, Brazil

**Keywords:** 6-OHDA, creatine, phosphocreatine, PI3K, neuroprotective, oxidative stress

## Abstract

Creatine is the substrate for creatine kinase in the synthesis of phosphocreatine (PCr). This energetic system is endowed of antioxidant and neuroprotective properties and plays a pivotal role in brain energy homeostasis. The purpose of this study was to investigate the neuroprotective effect of creatine and PCr against 6-hydroxydopamine (6-OHDA)-induced mitochondrial dysfunction and cell death in rat striatal slices, used as an *in vitro* Parkinson’s model. The possible involvement of the signaling pathway mediated by phosphatidylinositol-3 kinase (PI3K), protein kinase B (Akt), and glycogen synthase kinase-3β (GSK3β) was also evaluated. Exposure of striatal slices to 6-OHDA caused a significant disruption of the cellular homeostasis measured as 3-(4,5 dimethylthiazol-2-yl)-2,5-diphenyl-tetrazolium bromide reduction, lactate dehydrogenase release, and tyrosine hydroxylase levels. 6-OHDA exposure increased the levels of reactive oxygen species and thiobarbituric acid reactive substances production and decreased mitochondrial membrane potential in rat striatal slices. Furthermore, 6-OHDA decreased the phosphorylation of Akt (Serine^473^) and GSK3β (Serine^9^). Coincubation with 6-OHDA and creatine or PCr reduced the effects of 6-OHDA toxicity. The protective effect afforded by creatine or PCr against 6-OHDA-induced toxicity was reversed by the PI3K inhibitor LY294002. In conclusion, creatine and PCr minimize oxidative stress in striatum to afford neuroprotection of dopaminergic neurons.

## Introduction

Parkinson’s disease (PD) is the second most common neurodegenerative disease associated to aging (Lo Bianco et al., 2004). Animal and cell models used to study the pathological mechanisms underlying neurodegeneration in PD often involve the administration of toxins that selectively destroy or interrupt the activity of nigrostriatal dopaminergic neurons. These models include the treatment with reserpine, 6-hydroxydopamine (6-OHDA), or 1-methyl-4-phenyl-1,2,3,6-tetrahydropyridine (MPTP), which have contributed to the understanding of neurodegeneration in PD (Carlsson et al., 1957; Ungerstedt, 1971; Langston et al., 1984).

6-OHDA was first isolated in the 1950s by Adachi et al. (1964), and Ungerstedt (1971) was the first to use this compound to cause injury in the nigrostriatal dopaminergic pathway in rats nearly 50 years ago. Nowadays, 6-OHDA is widely employed for both *in vitro* and *in vivo* PD research (Valette et al., 1972; Roeling et al., 1995; Annett et al., 1997). This neurotoxin shows high affinity for the dopamine transporter (Lehmensiek et al., 2006), and once inside the neuron, it accumulates and undergoes nonenzymatic auto-oxidation, promoting reactive oxygen species (ROS) formation (Blandini et al., 2008) and selective damage of dopaminergic/catecholaminergic neurons. Because 6-OHDA induces ATP depletion (Tirmenstein et al., 2005), it has been hypothesized that mitochondrial dysfunction is related to cell death induced by 6-OHDA (Tobon-Velasco et al., 2013). Based on this evidence, one may propose that agents that improve cellular bioenergetics could reverse this neurodegenerative process.

The creatine kinase/phosphocreatine (PCr) system has been reported to play a complex and multifaceted role in brain energy homeostasis by maintaining high ADP levels at the site ATP is generated in the mitochondria and low levels at the site of ATP utilization (Bessman and Carpenter, 1985; Wyss et al., 1992). In brain cells, ATP levels are regulated by the cytosolic brain-specific isoform of creatine kinase (BB-CK) along with the mitochondrial isoform (ubiquitous mitochondrial creatine kinase, uMT-CK) and their substrates creatine and PCr, respectively (Hemmer and Wallimann, 1993). In the caudate, putamen, and midbrain of PD patients, a bilateral reduction of high-energy phosphates such as ATP and PCr is reported, suggesting that compounds that increase the high-energy phosphates could reverse neurodegeneration in PD (Hattingen et al., 2009). For example, oral creatine supplementation attenuates l-DOPA-induced dyskinesia in 6-OHDA-lesioned rats (Valastro et al., 2009).

Creatine has shown neuroprotective properties in different preclinal models (Matthews et al., 1999; Andres et al., 2005a, 2005b; Hosamani et al., 2010; Yong-Kee et al., 2011; Cunha et al., 2013); there are studies that ascribed this effect to its capacity to buffer cellular ATP levels coupled with mitochondrial targeted antioxidant properties in cell and mammalian models (Lensman et al., 2006; Sestili et al., 2006). Although the reversible conversion of creatine and ATP into PCr and ADP by mitochondrial creatine kinase to generate highly diffusible PCr energy reserves is an important element in cellular homeostasis, creatine also presents pleiotropic effects, as it modulates mitochondrial dysfunction, possesses antioxidant properties, and can inhibit the opening of the mitochondrial permeability transition pore (Lawler et al., 2002; Dedeoglu et al., 2003; Rambo et al., 2013). It remains to be established if these other mechanisms of actions can contribute to its neuroprotective effect against 6-OHDA-induced cell death.

As a product of the creatine kinase reaction, PCr accumulates within the cell in high concentrations. Several studies have reported the enigmatic protective cardiac effect of PCr in cardioplegic solutions (Sharov et al., 1986, 1987; Ruda et al., 1988), as PCr is unlikely to cross plasma membranes. However, a recent study shows that PCr can directly bind to phospholipid-containing membranes with low affinity, alters structural and conformational parameters of phospholipid liposomes, and protects phospholipid liposomes and erythrocytes from permeabilization induced by melittin, doxorubicin, hypoosmotic stress, or saponin (Tokarska-Schlattner et al., 2012). These results suggest that the interaction between PCr and membrane phospholipids may not only protect cellular membranes against various insults but could also have implications for many physiological membrane-related functions that are relevant for health and disease. For example, rats pretreated with PCr and exposed to focal cerebral ischemia injury had better neurologic scores, less infarction volume, fewer ultrastructural histopathologic changes, lower thiobarbituric acid reactive substances (TBARS) levels, and reduced apoptosis as compared with control group (Rauchova et al., 2002; Li et al., 2011).

The positive results obtained with creatine in experimental studies prompted its use in clinical trials in PD patients. In a pilot trial, creatine supplementation improved mood and led to a smaller dose increase of dopaminergic therapy in PD patients (Bender et al., 2006). Furthermore, PD rate progression was slowed by almost 50% at 1 year in the creatine-treated patients (Investigators NN-P, 2006). Although PCr has shown antiarrhythmic effects in patients with acute myocardial infarction (Ruda et al., 1988), there is scarce data regarding its potential effect in neurodegenerative diseases.

In this context, this study was designed to determine the possible neuroprotective and antioxidant actions of creatine and its product PCr in rat striatal slices exposed to the neurotoxin 6-OHDA, as an *in vitro* model of PD. The main advantages of using striatal slices, instead of cell cultures, is that they can be obtained from adult rodent brain, the pattern of synaptic connections within the slice is minimally altered, neuron-astrocyte-microglia interactions are preserved, and they contain the molecular machinery that allows them to maintain dopamine homeostasis and intact architecture of the dopamine terminal fields for several hours (Somjen et al., 1987). Our results indicate that both creatine and PCr are capable of providing neuroprotection of striatal slices exposed to the dopaminergic toxin 6-OHDA.

## Materials and Methods

### Materials

The following drugs and reagents were used: creatine monohydrate, dimethyl sulfoxide (DMSO), LY23390, *N*-Acetylcysteine (NAC), PCr (all from Sigma Chemical Company, St Louis, MO, USA), and Dulbecco’s modified Eagle’s medium (DMEM, Gibco).

### Animals

All experiments were performed using adult male Sprague-Dawley rats (275–325 g) from a colony of our animal quarters. The experiments were performed after approval of the protocol by the institutional Ethics Committee, in accordance with the European guidelines for the use and care of animals for research in accordance with the European Union Directive of September 22, 2010 (2010/63/UE) and with the Spanish Royal Decree of February 1, 2013 (53/2013). All efforts were made to minimize animal suffering and to reduce the number of animals used in the experiments.

### Striatal Slices Preparation

Rats were decapitated under sodium pentobarbital anesthesia (60 mg/kg, ip), forebrains were rapidly dissected and placed into ice-cold Krebs bicarbonate dissection buffer (pH 7.4), containing (in mM) NaCl 120, KCl 2, CaCl_2_ 0.5, NaHCO_3_ 26, MgSO_4_ 10, KH_2_PO_4_ 1.18, glucose 11, and sucrose 200. The chamber solutions were prebubbled with 95% O_2_/5% CO_2_ gas mixture for at least 45 min before slice immersion, to ensure O_2_ saturation. Thereafter, corpus of striatum was dissected and sectioned in transverse slices of 350 µm (for MTT assay) or 200 µm (for fluorescence and lactate dehydrogenase [LDH] assays) thick using a McIlwain Tissue Chopper (The Mickle Laboratory Engineering Co. Ltd., Gomshall, England). After an initial stabilization period of 30 min, slices were incubated in Krebs bicarbonate buffer (KRB) with the following composition (in mM): NaCl 120, KCl 2, CaCl_2_ 2, NaHCO_3_ 26, MgSO_4_ 1.19, KH_2_PO_4_ 1.18, and glucose 11 gassed with 95% O_2_/5% CO_2_. After preincubation in KRB, striatal slices were maintained in a nutritive incubation medium composed of 50% of KRB, 50% DMEM, 20 mM HEPES, and 100 µg/ml gentamicin, at 37°C in a CO_2_ atmosphere, as used in organotypic slices cultures (Gahwiler, 1987; Gahwiler et al., 1997) and previously adapted and validated to tissue slices by Molz et al. (2008).

### Slices Treatment

To investigate the concentration-dependent effects of 6-OHDA on cell viability, slices were exposed for 4 h to 6-OHDA (50–300 µM) diluted in the incubation medium described earlier.

To determine the potential neuroprotective effect of creatine or PCr on the toxicity induced by 6-OHDA, these compounds were coincubated with 6-OHDA for 4 h. Moreover, aiming at investigating the involvement of phosphatidylinositol-3 kinase (PI3K) activation in the protective effect of creatine or PCr, parallel experiments were performed in the presence of 30 µM LY-294002, which was added to the medium 30 min before and throughout cotreatment with 6-OHDA plus creatine or PCr.

### Evaluation of Striatal Slices Viability (MTT Assay)

Striatal slice homeostasis (metabolic function) was determined through the ability of the cells to reduce MTT (Mosmann, 1983). After treatments, striatal slices were incubated with MTT (0.5 mg/ml) in KRB solution for 30 min at 37°C. The tetrazolium ring of MTT can be cleaved by active dehydrogenases to produce a precipitated formazan salt. The formazan was solubilized by adding DMSO which gave a colored compound whose optical density was measured at 540 nm.

### Evaluation of Cell Membrane Damage as LDH Release

After treatments, plasma membrane integrity in striatum slices was monitored by measuring LDH released into the incubation media (Sobrado et al., 2004) using a commercial kit (Cytotoxicity Detection Kit, Roche) and following the manufacturer’s indications.

### Measurement of ROS Production in Striatal Slices

The molecular probe 2′,7′-dichlorodihydrofluorescein diacetate (H_2_DCFDA) that diffuses through the cell membrane and is hydrolyzed by intracellular esterases to the nonfluorescent form 2′,7′-dichlorofluorescein (DCFH) was used. DCFH reacts with intracellular ROS (such as H_2_O_2_) to form dichlorofluorescein (DCF), a green fluorescent dye. DCF fluorescence intensity is proportional to the amount of ROS. The measurement of ROS formation was based on the protocols standardized by Ali et al. (1992), with few modifications (Colle et al., 2012). After treatments, striatal slices were homogenized, and an aliquot of 20 µL of the homogenate was used for protein determination. H_2_DCFDA (5 µM) was added to supernatants, and fluorescence was read after 30 min using excitation and emission wavelengths of 480 and 525 nm, respectively. ROS levels, expressed as nmol of oxidized DCF per mg protein, were calculated by interpolation in a standard curve of oxidized DCF (constructed in parallel), corrected by the content of protein per sample and expressed as percentage of DCF oxidized formed versus the control values. Data from five experiments per group were collected and analyzed.

Additionally, the estimation of reactive ROS formation in the putamen and globus pallidus was carried out, as described by Egea et al. (2012). Immediately after chopper sectioning, 200 -µm-thick striatal slices were loaded with 80 μM H_2_DCFDA for 45 min in Krebs solution (120 mM NaCl, 2 mM KCl, 2 mM CaCl_2_, 26 mM NaHCO_3_, 1.19 mM MgSO_4_, 1.18 mM KH_2_PO_4_, and 11 mM glucose). Subsequently, the slices were washed twice with Krebs solution, and the protocol was started. Fluorescence was measured in a fluorescence-inverted NIKON eclipse T2000-U microscope (Nikon Instruments Europe, Badhoevedorp, the Netherlands). Wavelengths of excitation and emission were 485 and 520 nm, respectively. Images were taken at globus pallidus and putamen at magnifications of 100×. Fluorescence analysis was performed using the Metamorph Program version 7.0. Fluorescence in basal conditions was taken as 100%, and experimental variables were normalized with respect to this value.

### Lipid Peroxidation Assay

Lipid peroxidation was assessed in homogenates obtained from the striatal slices (four slices per test) by the assay of TBARS formation, according to previous reports (Rios and Santamaria, 1991; Colle et al., 2012). Immediately, after the last incubation, slices were homogenized in 500 µL of ultrapurified water and an aliquot of 20 µL of the homogenate was separated for protein determination. The remaining homogenates were mixed with 1 mL of the TBA reagent (containing 15% of trichloroacetic acid, 0.375% of thiobarbituric acid, and 2.5%, v/v of HCl) to be reincubated in a boiling water bath (95°C) for 30 min. Samples were then centrifuged at 3,000 × g for 15 min. The optical density of supernatants was estimated at 540 nm. The concentrations of MDA (expressed as nmol of MDA per mg protein) were calculated by interpolation in a standard curve of MDA (constructed in parallel), corrected by the content of protein per sample and expressed as percent of MDA formed versus the control values. Data from five experiments per group were collected and analyzed.

### Studies on 6-OHDA Autoxidation

The autoxidation of 6-OHDA was followed spectrophotometrically by monitoring the formation of *p*-quinone at 490 nm (Sachs et al., 1975; Soto-Otero et al., 2000). For each assay, 300 µl of nutritive culture medium was incubated in a microplate at 37°C. Then, the autoxidation was initiated with addition of 6-OHDA at a final concentration of 0.5 mM. The monitoring of kinetics was immediately initiated and maintained for the subsequent 5 min.

### Measurement of Mitochondrial Membrane Potential

Slices were loaded with the mitochondrial selective ﬂuorescent dye, tetramethylrhodamine ethyl ester (TMRE, 3 µM) for 15 min at 37°C (Egea et al., 2007; Dal-Cim et al., 2013). Then, they were washed three times with KRB. Finally, ﬂuorescence was measured in a ﬂuorescence-inverted NIKON eclipse T2000-U microscope. Wavelengths of excitation and emission for TMRE (mitochondrial membrane potential) were 550 and 590 nm, respectively. Images were taken at the putamen and globus pallidus at magniﬁcations of 100×. Fluorescence analysis was performed using the Metamorph program version 7.0 (Molecular Devices, LLC, Sunnyvale, CA, USA). Fluorescence was measured in three different areas within the same striatum region to obtain the average value. Average ﬂuorescence in basal conditions was taken as 100%, and experimental variables were normalized with respect to this.

### Western Blot Analysis

Striatal slices were lysed in 100 μl ice-cold lysis buffer (1% Nonidet P-40, 10% glycerol, 137 mM NaCl, 20 mM Tris–HCl, pH 7.5, 1 µg/ml leupeptin, 1 mM phenylmethylsulfonyl fluoride, 20 mM NaF, 1 mM sodium pyrophosphate, and 1 mM Na_3_VO_4_). A tablet of protease inhibitor cocktail (complete Mini, Roche) was added for each 10 ml of buffer. Protein concentrations were measured according to the method described by Lowry et al. (1951), using bovine serum albumin as a standard. Equivalent amounts of protein were electrophoresed in 10% sodium dodecyl sulfate denaturing polyacrylamide slab gels. After transfer to an Immobilon-P Transfer Membrane (Millipore, Bedford, MA, USA) at room temperature, membranes were blocked in Tris-buffered saline with 0.05% Tween 20 (TBST) containing 5% of albumin, and incubated for 2 h at room temperature with the primary antibody against p-Akt, protein kinase B (Akt), p-GSK3β, glycogen synthase kinase-3β (GSK3β), tyrosine hydroxylase (TH), or β-actin (1:1,000; Santa Cruz Biotechnology Inc., Santa Cruz, CA, USA) and incubated for 1 h with secondary antibodies conjugated with peroxidase (1:10,000). The immunoblots were developed using enhanced chemiluminescence reagents (Amersham Biosciences). Optical density was quantified using the program Scion Image® Alpha 4.0.3.2. Control conditions were taken as 100% and experimental variables were normalized with respect to this value.

### Data Analysis

Data are represented as means + *SEM*. Comparisons among experimental and control groups were performed by one-way ANOVA followed by the Newman-Keuls post hoc test. Statistical difference was accepted when *p* ≤ .05.

## Results

### 6-OHDA Induces Cell Death in the Striatal Slices

**Figure 1.**
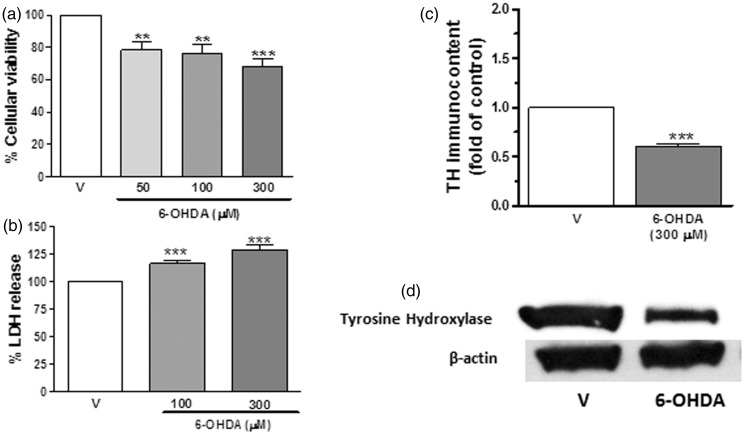
6-OHDA induces cell dyshomeostasis in striatal slices. Cell viability measured as MTT reduction (a), lactate dehydrogenase release (b), or TH immunocontent (c) in rat striatal slices after 4 h exposure to 6-OHDA (50–300 µM). Panel (d) shows a representative immunoblotting for TH and β-actin. Each column represents the mean + *SEM* of five to seven experiments. **p < .01, ***p < .001 compared with vehicle-treated slices.

6-OHDA is one of the most common neurotoxins used in *in vivo* and *in vitro* neurodegenerative models of central catecholaminergic projections, including the nigrostriatal system (Ungerstedt, 1968, 1971, 1976; Sachs et al., 1975; Blum et al., 2001). In the present study, rat striatal slices exposed to 6-OHDA, in the range of 50 to 300 μM, for 4 h significantly decreased cell viability in a concentration-dependent manner; the survival rate for MTT analyses was 78.6% at 50 μM and 68.8% at 300 μM 6-OHDA (Figure 1(a)). Furthermore, this toxic compound significantly increased cell membrane damage; LDH release rates were 116.6% at 100 μM and 129.0% at 300 μM (Figure 1(b)). 6-OHDA, at the concentration of 300 µM, also induced a significant cell dyshomeostasis in striatal slices represented as a significant reduction of TH levels (60%; Figure 1(c)). Based on these results, we selected 300 µM 6-OHDA for the following experiments.

### Creatine and PCr Afford Neuroprotection Against 6-OHDA-Induced Cell Death in Striatal Slices

**Figure 2.**
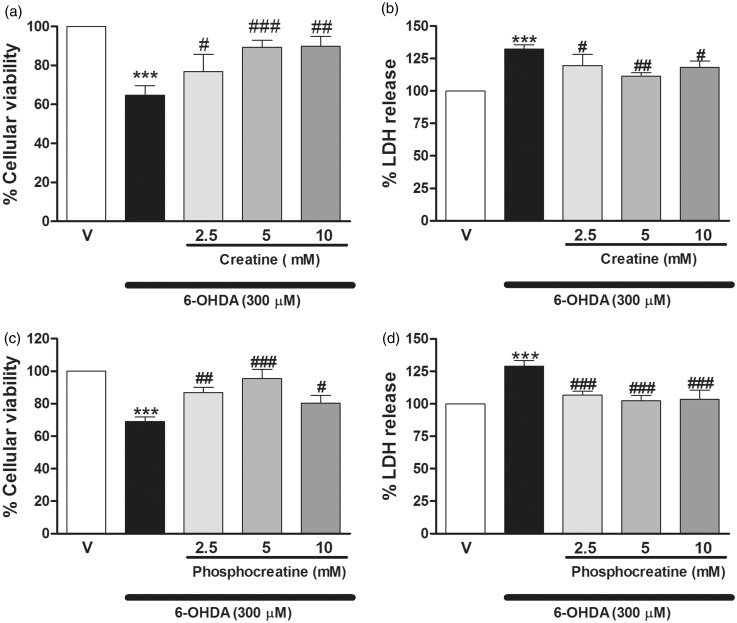
Creatine and PCr afford neuroprotection in striatal slices exposed to 6-OHDA. MTT reduction (a and c, respectively) or LDH activity (b and d, respectively) was analyzed 4 h after 6-OHDA addition. Coincubation (4 h during 6-OHDA incubation) of the rat striatal slices with creatine (2.5–10 mM) or PCr (2.5–10 mM) increased cell viability (a and c, respectively) and decreased the LDH release (b and d, respectively) as compared with the group incubated with 6-OHDA alone. Each column represents the mean + *SEM* of six to eight experiments. ***p < .001 compared with the vehicle-treated basal. ^#^*p* < .05, ^##^*p* < .01, ^###^*p* < .001 compared with 300 µM 6-OHDA group.

Concentrations of 2.5 to 10 mM of creatine or PCr were chosen in this study based on previous studies that demonstrated neuroprotective actions of creatine, at the milimolar range, against MPP^+^ or rotenone cytotoxicity (Andres et al., 2005b; Yong-Kee et al., 2011). Cells cotreated with various concentrations of creatine or PCr (2.5–10 mM) plus 6-OHDA (300 µM) for 4 h presented a significant increase on survival rate, measured as MTT metabolism, in relation to the 6-OHDA group alone. Significant protection of creatine and PCr was achieved at 2.5 mM (12.2% and 17.8% increase in cell survival, as compared with the 6-OHDA group), and maximum protection was achieved at a concentration of 5 mM (24.6% and 26.4% increase in cell survival; Figure 2(a) and (c)). Furthermore, creatine and PCr at concentrations of 2.5 to 10 mM attenuated 6-OHDA-induced increase in LDH in striatal slices; significant protection of creatine and PCr was achieved at 2.5 mM (12.7% and 22.4% decrease LDH release, respectively, as compared with the 6-OHDA group). Maximum protection was obtained at a concentration of 5 mM (20.8% and 26.6% increase in cell survival; Figure 2(b) and (d)). Considering that the concentration of 5 mM of creatine and PCr exerted maximum protection against the toxic insult, we selected this concentration to evaluate their mechanism of action.

### Creatine or PCr Do Not Inhibit 6-OHDA Auto-Oxidation

**Figure 3.**
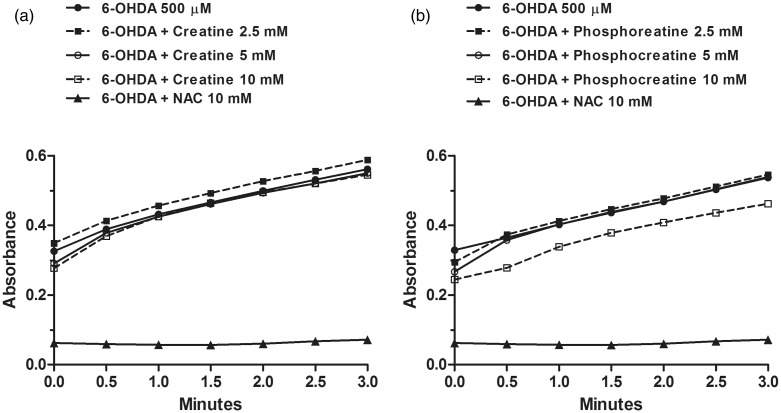
Creatine or PCr do not inhibit 6-OHDA auto-oxidation. The autoxidation of 6-OHDA in a nutritive incubation medium was monitored during 3 min. Neither creatine (5 mM) nor PCr (5 mM) blocked 6-OHDA auto-oxidation. The positive control NAC (10 mM) prevented the 6-OHDA auto-oxidation. Data are represented as mean of three experimental determinations.

6-OHDA toxicity has been shown to be directly correlated to its auto-oxidation rate (Graham et al., 1978; Soto-Otero et al., 2000). Therefore, we spectrophotometrically monitored the autoxidation of 6-OHDA in a nutritive incubation medium to investigate the potential scavenger effects of creatine or PCr. As shown in Figure 3, there was a rapid increase in the absorbance at 490 nm following rectangular hyperbolic kinetics, indicating dopamine autoxidation. The initial rate for this process was estimated at 75.53 × 10^−3 ^± 4.50 × 10^−3^ ΔA/min. The corresponding solution manifested a rapid formation of red chromophores which after 24 h changed to an insoluble black pigment. The presence of 10 mM NAC (a positive control) significantly inhibited the increase in the absorbance at 350 nm, and the solution remained colorless for 14 days. Creatine or PCr (2.5–10 mM) did not change the rate of 6-OHDA autoxidation at 490 nm following a rectangular hyperbolic kinetics, with slopes of 76.61 × 10^− 3 ^± 4.71 × 10^−3^, 80.69 × 10^−3^ ± 8.14 × 10^−3^, and 84.04 × 10^−3 ^± 9.92 × 10^−3^ (creatine); and 78.31 × 10^−3 ^± 5.03, 83.39 × 10^−3 ^± 8.03, and 74.2 × 10^−3 ^± 5.51 (PCr) ΔA/min, respectively.

### Creatine and PCr Inhibit 6-OHDA-Induced Elevation of Intracellular ROS in Striatal Slices

**Figure 4.**
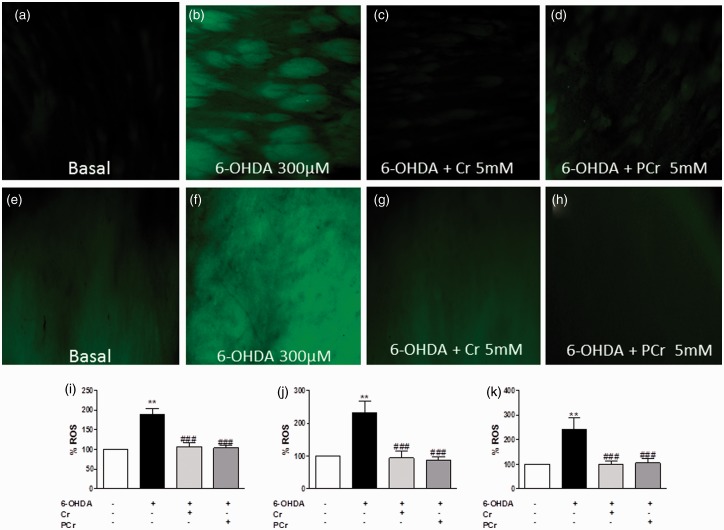
Creatine and PCr inhibit oxidative stress induced by 6-OHDA in rat striatal slices. ROS generation was estimated with the fluorescent probe, 2′,7′-dichlorofluorescein diacetate (H_2_DCFDA). DCF fluorescence images in the putamen (a–d) and globus pallidus (e–h) under the different experimental conditions indicated in each panel taken at 100×. Quantification of DCF fluorescence on rat striatal slices homogenates was measured in a Tecan microplate reader and expressed as nmol of oxidized DCF per mg protein and normalized to control 100%. The quantification of DCF fluorescence in the putamen (j) and globus pallidus (k) was measured in an epifluorescence NIKON eclipse T2000-U microscope. Quantification of the mean fluorescence obtained under each experimental condition in putamen and globus pallidus is normalized respect to control (100%). Each column represents the mean + *SEM* of six to nine experiments. **p < .01 compared with the basal group. ^###^*p* < .01 as compared with 300 µM 6-OHDA group.

Oxidative stress contributes to the cascade leading to dopamine cell degeneration in PD (Jenner, 2003). 6-OHDA induced ROS formation in the homogenate of striatal slices and also in putamen and globus pallidus. In a first set of experiments, striatal slices cotreated with creatine (5 mM) or PCr (5 mM) plus 6-OHDA (300 µM) for 4 h presented a decreased ROS formation, in comparison to the 6-OHDA group (Figure 4(i)). To analyze specific nuclei of striatum, the striatal slices were subjected to same experimental neurotoxicity/neuroprotection paradigm, and ROS generation was analyzed by fluorescence microscopy, as shown in the photomicrographs of the putamen (Figure 4(a) to (d)) and globus pallidus (Figure 4(e) to (h)). Fluorescence analysis indicated that creatine (5 mM) or PCr (5 mM) prevented the increase in ROS formation caused by 6-OHDA (300 µM) in putamen (Figure 4(j)) and globus pallidus (Figure 4(k)).

### Creatine and PCr Abolished 6-OHDA-Induced Mitochondrial Membrane Potential Changes in putamen and globus pallidus in Rat Striatal Slices

**Figure 5.**
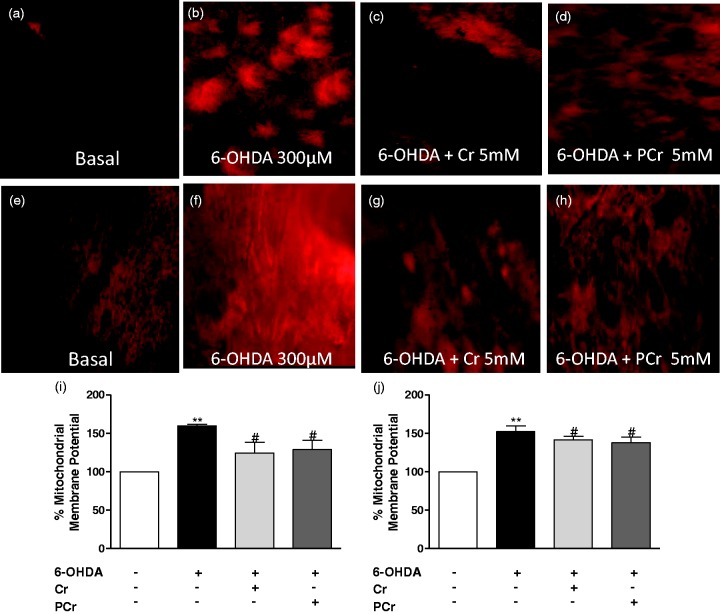
Creatine or PCr prevent loss of mitochondrial membrane potential in the rat striatal slices subjected to 6-OHDA. TMRE fluorescence in the putamen (a–d) and globus pallidus (e–h) was analyzed 4 h after coincubation with 6-OHDA plus creatine or PCr, as detailed in the Materials and Methods section. The quantification of TMRE fluorescence was measured in an epifluorescence NIKON eclipse T2000-U microscope (i, j). The top part of the figure illustrates representative photomicrographs of putamen and globus pallidus at 100×. Quantification of the mean fluorescence obtained under each experimental condition in putamen and globus pallidus is normalized respect to control (100%). Each column represents the means + *SEM* of six to eight experiments carried out in triplicates. **p < .01 compared with the basal group. ^#^*p* < .05 compared with 300 µM 6-OHDA group.

There is accumulating evidence from *in vitro* and *in vivo* studies suggesting that mitochondrial abnormalities are a common event in PD (Langston et al., 1984; Bindoff et al., 1989; Parker et al., 1989; Abou-Sleiman et al., 2006). Considering that mitochondrial membrane potential directly affects mitochondrial activity, we monitored this parameter in our experimental paradigm. As shown in Figure 5, 6-OHDA increased TMRE fluorescence in putamen (Figure 5(b) and (i)) and globus pallidus (Figure 5(f) and (j)) as an indication of mitochondrial depolarization. Under these experimental conditions, creatine or PCr (5 mM) mitigated mitochondrial depolarization caused by 6-OHDA in the putamen (Figure 5(c), (d) and (i)) and globus pallidus (Figure 5(g), (h) and (j)).

### Involvement of PI3K/Akt/GSK-3β in the Neuroprotective and Antioxidant Effect of Creatine and PCr

**Figure 6.**
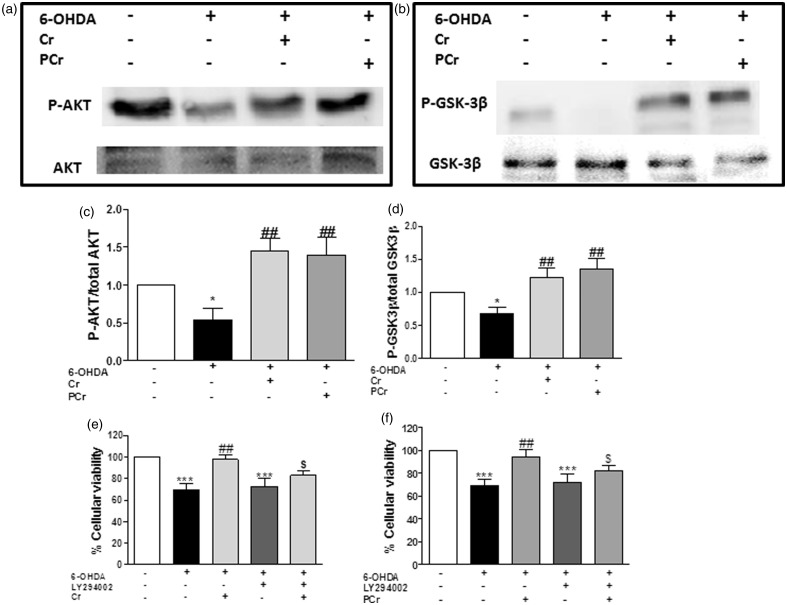
Protection afforded by creatine and PCr against 6-OHDA-induced toxicity in rat striatal slices is associated with Akt (Serine^473^) and GSK3β (Serine^9^) phosphorylation. Representative immunoblotting demonstrates Akt phosphorylation (a) and GSK3β phosphorylation (b). The level of Akt or GSK3β phosphorylation determined as a ratio of optic densitometry of the phosphorylated band over the optic densitometry of the total band (c and d, respectively) are expressed as percentage of the control. In another set of experiments, rat striatal slices were preincubated with 10 µM LY294002, 1 h before and during 6-OHDA plus creatine or PCr incubation (4 h). The protective effect of creatine or PCr on 6-OHDA-induced cell death was prevented by LY294002 (e and f, respectively). Each column represents the mean + *SEM* of four to six animals. ^*^p < .05, ^***^p < .001, when compared with control. ^##^*p* < .01 as compared with 300 µM 6-OHDA group. ^$^*p* < .05, compared with 300 µM 6-OHDA plus creatine or PCr group.

Significant attention has been given to the potential role of defective PI3K/Akt signaling in PD neurodegeneration and to the possibility that activation of Akt may provide neuroprotection in this neurodegenerative disease (Timmons et al., 2009). We therefore investigated whether PI3K/Akt/GSK3β signaling is involved in the neuroprotective and antioxidant effect found for creatine and PCr against 6-OHDA-induced neurotoxicity in striatal slices. We first found that 6-OHDA induced a significant decreased in Akt and GSK3β phosphorylation and that creatine and PCr suppressed 6-OHDA-induced phosphorylation dysfunctions (Figure 6(c) and (d), respectively).

The participation of the PI3K/Akt pathway was further demonstrated when we found that the protective actions of creatine and PCr were abolished in the presence of the PI3K inhibitor LY294002 (Figure 6(e) and (f)).

**Figure 7.**
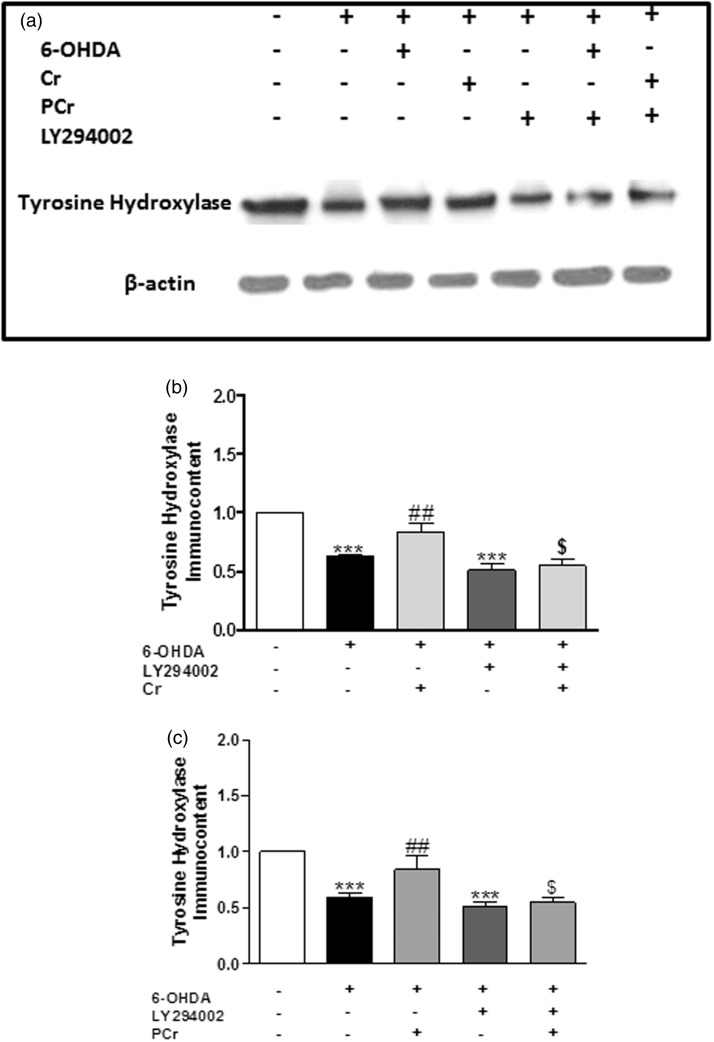
Involvement of PI3K/Akt in the protective effect of creatine and PCr on TH levels. Rat striatal slices were preincubated with 10 µM LY294002 1 h before and during 6-OHDA plus creatine or PCr incubation (4 h). (a) Representative immunoblotting of tyrosine hydroxylase is shown. The TH immunocontent determined as a ratio of optic densitometry of the TH band over the optic densitometry of the β-actin band are expressed as percentage of the control. Creatine (5 mM) or PCr (5 mM) prevented the decrease of TH levels induced by 6-OHDA in the rat striatal slices, and these effects are significantly abolished by LY294002 (b and c, respectively). Each column represents the mean + *SEM* of four to six animals. ^***^p < .001, when compared with control. ^##^*p* < .01 compared with 300 µM 6-OHDA group. ^$^*p* < .05 compared with 300 µM 6-OHDA plus creatine or PCr group.

Data from literature show that total Akt and p-Akt (Serine^473^) is found at high levels in TH immunopositive dopaminergic neurons in human brain, and selective loss of these neurons is accompanied by a marked decrease of total Akt and p-Akt (Serine^473^) levels in the PD brain (Timmons et al., 2009). We therefore considered of interest to investigate how LY294002 could modify the ability of creatine and PCr to restore TH levels in striatal slices exposed to 6-OHDA. As shown in Figure 7(b) and (c), the presence of LY294002 (30 µM) abolished the capacity of creatine and PCr to restore TH levels in striatal slices exposed to 6-OHDA.

**Figure 8.**
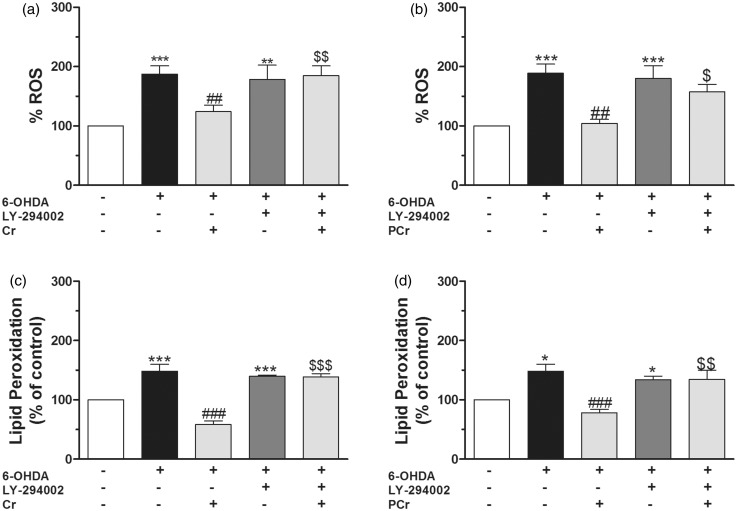
Creatine and PCr decrease ROS production and lipid peroxidation in rat striatal slices subjected to 6-OHDA via PI3K/Akt activation. Rat striatal slices were preincubated with 10 µM LY294002 1 h before and during 6-OHDA plus creatine or PCr incubation (4 h). Quantification of DCF fluorescence on the rat striatal slices homogenates was measured in a Tecan microplate reader (expressed as nmol of oxidized DCF per mg protein and normalized to control 100%). The concentrations of MDA on the rat striatal slices homogenate were expressed as nmol of MDA per mg protein and were calculated by interpolation in a standard curve of MDA, corrected by the content of protein per sample and expressed as percent of MDA formed versus the control values. LY294002 abolished the antioxidant effects of creatine and PCr on ROS production (a and b, respectively) and lipid peroxidation (c and d, respectively) induced by 6-OHDA. ^*^p < .05, ^**^p < .01,^***^p < .001 when compared with control. ^##^*p* < .01, ^###^*p* < .001 compared with 300 µM 6-OHDA group. ^$^*p* < .05, ^$$^*p* < .01, ^$$$^*p* < .001 as compared with 300 µM 6-OHDA plus creatine or PCr group.

Because antioxidant compounds activate Akt and exert a protective effect (Toth et al., 2003; Latha et al., 2013; Sui et al., 2014), we investigated whether PI3K/Akt signaling was involved in the effect of creatine or PCr against 6-OHDA-induced oxidative stress in striatal slices. Creatine and PCr suppressed 6-OHDA-induced increase in ROS formation (Figure 8(a) and (b), respectively) and TBARS levels (Figure 8(c) and (d), respectively). Under these experimental conditions, LY394002 blocked the antioxidant effect of creatine and PCr.

Taken together, the above results clearly show the participation of the PI3K/Akt pathway in the protective actions of creatine and PCr in striatal slices exposed to the neurotoxin 6-OHDA.

## Discussion

The main finding of this study is that the guanidine compounds creatine and PCr can afford neuroprotection in an *in vitro* model of PD by a mechanism that implicates modulation of oxidative stress through the activation of the PI3K/Akt/GSK3β intracellular pathway.

6-OHDA can induce dopaminergic cell death by three main mechanisms: (a) ROS generation by intra or extracellular auto-oxidation; (b) hydrogen peroxide (H_2_O_2_) formation induced by monoamine oxidase activity, and (c) direct inhibition of the mitochondrial respiratory chain. These events lead to oxidative stress, decrease in cellular ATP availability (Tirmenstein et al., 2005), and thereby, to cell death (Blum et al., 2001). Likewise, in our experimental protocol, 6-OHDA caused increased levels of ROS and lipid peroxidation, mitochondrial depolarization, and toxicity of rat striatal slices.

Brain metabolism impairment has been identified as an important mechanism underlying the pathophysiology of PD (Shoffner et al., 1991; Berndt et al., 2013). The creatine kinase/PCr system controls brain energy homeostasis and has been implicated in several neurodegenerative conditions (Adhihetty and Beal, 2008); for example, in PD patients, total creatine kinase activity decreases and cytotoxic ROS increases (Janetzky et al., 1994; Aksenova et al., 1999). Also, in brains exposed to agents that promote free radical production, creatine kinase activity decreases (Burmistrov et al., 1992), suggesting that compounds that block creatine kinase inhibition induced by oxidative stress could be neuroprotective in diseases like PD. Interestingly, a recent study demonstrates that creatine levels are increased in the right striatum following 6-OHDA striatal lesions in rats, which is interpreted as an endogenous compensation mechanism to restore energy supply in response to 6-OHDA-induced injury. This observation suggests a key role of this guanidine-like compound against 6-OHDA toxicity (Gao et al., 2013) and has led to the hypothesis that agents that modulate the bioenergetic status, mainly the creatine-containing compounds, could reverse the neurodegenerative process induced by 6-OHDA.

Creatine has been widely used as an ergogenic aid to improve exercise performance in humans and also to ameliorate oxidative stress-mediated diseases (Persky and Brazeau, 2001; Wyss and Schulze, 2002; Andres et al., 2008). Creatine supplementation can protect neurons against neurotoxins *in vitro* (Brewer and Wallimann, 2000) and it can slow down the progression of certain neurodegenerative conditions, as assessed in experimental animal models (Andreassen et al., 2001; Hosamani et al., 2010; Cunha et al., 2012). Furthermore, there are clinical trials that indicate that creatine supplementation could be beneficial in diseases like Huntington’s, amyotrophic lateral sclerosis, depression, or PD (Mazzini et al., 2001; Roitman et al., 2007; Beal, 2011; Gualano et al., 2012). This study shows that both creatine and PCr can protect striatal slices against 6-OHDA-cell death, suggesting a putative neuroprotective action of these compounds in PD. In a recent study from our research group, creatine reversed 6-OHDA toxicity in SH-SY5Y cells, a human neuroblastoma cell line used as a neuronal cell model (Cunha et al., 2013). Of note, in the present study, we show that PCr also can provide a similar protective effect as creatine in a more complex model such as the striatal slice. Reinforcing this notion, a study reported that PCr boosted cell survival against rotenone-induced cell death in SH-SY5Y cells (Sawmiller et al., 2012). Furthermore, in the present study, creatine and PCr prevented the decrease in TH levels in striatal slices exposed to 6-OHDA. A similar effect has been reported in mesencephalic cells exposed to the toxin MPP^+^ and treated with creatine (Andres et al., 2005b). Our results imply that both creatine and PCr are beneficial for the survival of TH-immunoreactive neurons when encountering an oxidative stress environment.

Interestingly, uMT-CK- deficient mice show increased sensitivity to MPTP-induced dopamine depletion and loss of TH-stained neurons; creatine administered to these mice was able to increase brain concentrations of both creatine and PCr and to exert neuroprotective effects against MPTP toxicity. These results suggest the pleotropic effects of creatine by a mechanism independent on the enzyme activity (Klivenyi et al., 2004). Reinforcing this notion, in a *Drosophila melanogaster* model, it was shown that creatine can protect from oxidative stress, although insects, expressing arginine kinase instead or creatine kinase, are not able to synthesize PCr (Hosamani et al., 2010). The pleiotropic effects of creatine could comprise antioxidant effects and activation of signaling pathways, including Akt/PKB.

The antioxidant activity of creatine or PCr emerges as mechanism that is likely to play a supportive role in the creatine or PCr cytoprotection paradigm (Sestili et al., 2011). Several studies, most with creatine, corroborate this action. For example, Hosamani et al. (2010) showed that creatine reduced ROS production and other oxidative markers such as malondialdehyde and hydroperoxide induced by rotenone in *Drosophila melanogaster*. In another oxidative stress cytotoxicity model induced by H_2_O_2_, creatine coincubation recovered cell viability (Sestili et al., 2009, 2011; Sartini et al., 2012). Lawler et al. (2002) have reported a direct antioxidant effect of creatine in a cellular system against aqueous radical and reactive species ions; however, our results show that neither creatine nor PCr block the auto-oxidation of 6-OHDA. Therefore, the protective effect observed here with these two compounds does not seem to be related with a direct antioxidant effect. Interestingly, a study by Tokarska-Schlattner et al. (2012) reported changes in phospholipid bilayer properties by membrane-bound PCr and subsequently protection of lipid membranes against permeabilization and cell lysis, suggesting that the neuroprotective effect of PCr could be dependent, at least in part, on membrane stabilization. In this study, we provide evidence that both creatine and PCr reduce ROS production and lipid peroxidation caused by 6-OHDA, supporting the assumption that these compounds may reduce oxidative stress.

Mitochondria being one of the main organelles to contribute to ROS production, we evaluated the effects of 6-OHDA on mitochondrial membrane potential; under these conditions, both creatine and PCr were able to attenuate mitochondrial depolarization induced by 6-OHDA. Therefore, this mechanism could further contribute to control the redox imbalance in the striatal slices exposed to the toxin.

As to the possible intracellular signaling pathways implicated in the neuroprotective mechanism related to creatine and PCr, our results indicate that the survival pathway PI3K/Akt plays a key role. This conclusion derives from the following results: (a) both creatine and PCr prevented the decrease in Akt phosphorylation at Serine^473^ induced by 6-OHDA, and (b) LY294002, a PI3K/Akt inhibitor, blocked the protective, the antioxidant, and recovery of TH levels afforded by creatine and PCr in slices exposed to 6-OHDA. In line with these results, a previous study showed that creatine increased Akt activity in C2C12 cells, a mouse myoblast cell line (Deldicque et al., 2007), and afforded neuroprotection via PI3K/Akt in the human neuroblastoma SH-SY5Y cell line (Cunha et al., 2013).

We have also investigated the effect of creatine and PCr on GSK3β; this serine/threonine kinase was originally identified as a regulator of glycogen metabolism but is now recognized as an important modulator of apoptosis. The inactivation of GSK3 can be induced by phosphorylation at one of its *N*-terminal serine residues: Serine^21^ for GSK3α and Serine^9^ for GSK3β (Plyte et al., 1992). The administration of agents that cause phosphorylation and consequent inactivation of GSK-3β is considered an interesting pharmacologic neuroprotective strategy. Phosphorylation of GSK3 can be mediated by several kinases, including Akt (Rommel et al., 1999; Jope and Roh, 2006). Literature data have reported that pretreatment with TDZD-8, lithium, or L803-mts (GSK3 inhibitors) reduces 6-OHDA-induced cell death (Chen et al., 2004), and knockdown of GSK3β attenuates 6-OHDA-induced apoptosis in SH-SY5Y cells (Li et al., 2011). In the present study, we have observed that 6-OHDA decreases GSK3β Serine^9^ phosphorylation, and both creatine and PCr were able to reverse this effect.

In conclusion, our findings identify creatine and PCr as potent natural protective factors for dopaminergic cell survival; their neuroprotective mechanism does not seem to be related to a direct scavenger effect but to mitochondrial membrane stabilization, activation of the survival signaling pathway PI3K/Akt/GSK3β, and control of the redox balance.
